# 

**Published:** 1894-07-21

**Authors:** 


"Johannis" THE KING OF NATURAL TABLE WATERS. "Johannis"

### THE HOSPITAL

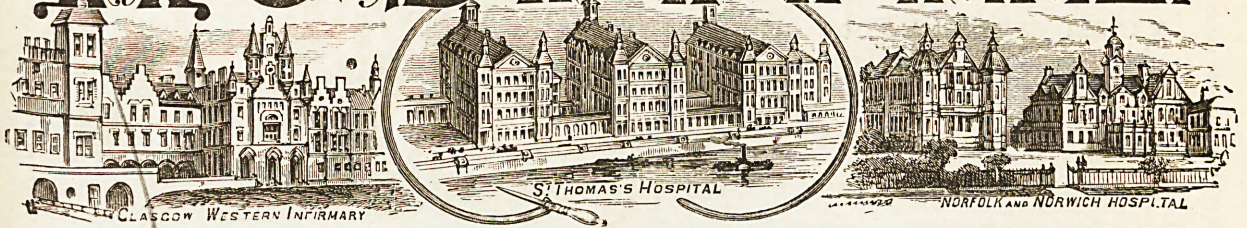

No. 408. Vol. XVI.
SATURDAY, JULY 21ST, 1894.

WITH EXTRA NURSING SUPPLEMENT.
[Registered at the General Post Office as a Newspaper for transmission in the United Kingdom and Abroad.]

PRICE TWOPENCE.

Per Annum, 10s. 6d., post free.

Monthly Parts, 6d.; post free, 8d.

Ditto per Annum, 8s.6d., post free.

